# Advantages in functional imaging of the brain

**DOI:** 10.3389/fnhum.2015.00249

**Published:** 2015-05-19

**Authors:** Walter Mier, Daniela Mier

**Affiliations:** ^1^Heidelberg University HospitalHeidelberg, Germany; ^2^Central Institute of Mental Health, Medical Faculty Mannheim/University of HeidelbergMannheim, Germany

**Keywords:** positron emission tomography, molecular imaging, radiotracers, neurological diseases, functional magnetic imaging

## Abstract

As neuronal pathologies cause only minor morphological alterations, molecular imaging techniques are a prerequisite for the study of diseases of the brain. The development of molecular probes that specifically bind biochemical markers and the advances of instrumentation have revolutionized the possibilities to gain insight into the human brain organization and beyond this—visualize structure-function and brain-behavior relationships. The review describes the development and current applications of functional brain imaging techniques with a focus on applications in psychiatry. A historical overview of the development of functional imaging is followed by the portrayal of the principles and applications of positron emission tomography (PET) and functional magnetic resonance imaging (fMRI), two key molecular imaging techniques that have revolutionized the ability to image molecular processes in the brain. We conclude that the juxtaposition of PET and fMRI in hybrid PET/MRI scanners enhances the significance of both modalities for research in neurology and psychiatry and might pave the way for a new area of personalized medicine.

## Principles of Diagnostic Imaging

Traditionally, diagnostic imaging was restricted to the visualization of aberrations in morphological structures. However, neurological pathologies generally do not cause significant correlates to macroscopically or microscopically visible changes in cell morphology. Since the contrast obtained for the differentiation of pathophysiological abnormality defines the performance of an imaging modality, highly sensitive methods for imaging alterations in brain functioning had to be developed. In general, any kind of radiation that penetrates the human body can be used as signal source for diagnostic imaging. Hence, the diversity of imaging modalities reflects the variety of the capabilities of the different kinds of radiation used in clinical practice. The aim of this short review is to introduce the principles of the most common and most promising molecular imaging techniques for the investigation of alterations in brain functioning: positron emission tomography (PET) and functional magnetic resonance imaging (fMRI) and to deduce their limitations and prospects.

The contemporary imaging methods: computer tomography (CT), magnetic resonance imaging (MRI), PET, and ultrasound (US) can be traced back to the German physicist Wilhelm Conrad Röntgen. In 1895 Röntgen recognized that X-rays penetrate solid mater. He further discovered that the attenuation of X-rays depends on the characteristics of the object penetrated. When he subsequently deduced the technique of X-ray imaging a great invention took place: the visualization of structures inside the body without using surgery!

Since then the imaging with X-rays has been shown to provide images of excellent anatomic resolution. The development of CT, the corresponding tomographic modality, has further enhanced X-ray imaging and up to the present billions of scans have been carried out. The specific strength of CT imaging is its high anatomic resolution. The imaging is straightforward as long as bony materials or calcified tissue is investigated. In soft tissues the differences of X-ray attenuation are marginal and contrast agents are used to provide the known high-quality CT images in the major areas of the body i.e., the vascular system, the lungs and the kidneys. Inclusion of elements of high atomic number is pivotal for the efficacy of CT contrast agents as the X-ray absorbency is almost directly proportional to the third power of an elements atomic number. However, unfortunately, most elements of high atomic number are not biocompatible and iodine containing contrast agents dominate the choice of contrast agents in clinical use. High doses of up to 42 g of the contrast agent are required to induce sufficient contrast (Mortelé et al., 2005). Hence, for brain imaging in particular the highly sensitive methods PET and MRI are more promising.

## Principles of Molecular Imaging

When it comes to the tasks faced in brain imaging, the assessment of cellular functions and the visualization of metabolic processes is required. The visualization of changes of essential cellular structures gives testimony about the health status of a cell long before macroscopically or microscopically visible changes in cell morphology occur. These sensitive phenomena are only accessible to investigations with tracers/imaging agents and in the most delicate cases extremely small numbers of receptors are available to trigger the specific accumulation of the imaging agent (Catana et al., 2012).

Generally, there are two types of imaging agents to be differentiated (a) passive agents that modulate an external signal i.e., in CT and US; and (b) probes that either produce a signal autonomously i.e., radiotracers and bioluminescent dyes, or transformations of an external signal, such as with MRI contrast agents or fluorescent probes. As passive contrast agents only enhance the body’s signal, relatively high doses are required to delineate their endogenous contrast. While very high concentrations of CT contrast agents are required, the signal of radiolabeled tracers can be measured at an exceptionally high sensitivity. As the radioactive signal is exclusively emitted by the endogenous tracer it can be definitively traced back to the imaging agent. The short lived positron emitters ^11^C and ^18^F are ideal for molecular imaging purposes. The positrons emitted by these radioisotopes produce gamma rays upon collision with electrons. These gamma rays efficiently penetrate the body and are detected in the PET scanner. The radioisotope is linked to a tracer molecule that triggers specificity by i.e., binding neuroreceptors or tracking the hemodynamics of the brain. Due to their pivotal function in neurotransmission and neuromodulation, the assessment of the spatial and temporal activity of neuroreceptors virtually depicts the brain functions and physiologic activities. Hence, PET tracers are the prototype of imaging agents for functional brain imaging, and PET studies have been proven of vital importance for the understanding and therapy of neurologic and psychiatric disorders.

## Applications for PET Tracers

Selective radioligands are often derived from psychoactive drugs and gain their specificity by mimicking neurotransmitters. The visualization of their specific binding to a target such as a receptor, a transporter or an enzyme allows revealing various pathologic conditions. In the following, PET tracers will be reviewed that target receptors, proteins or neurotransmitter, or glucose consumption.

Typical examples of radiotracers for PET are the ligands for the quantitative imaging of specific neuroreceptor subtypes such as [^11^C]raclopride and [^18^F]fallypride for dopamine D2/D3 receptors for differential diagnosis of movement disorders and for assessment of receptor occupancy by neuroleptics drugs in schizophrenia (Siessmeier et al., 2005). Tracers binding arterial nicotinic acetylcholine receptors and acetylcholinesterase such as [^18^F]-2-fluoro-A85380 have been developed as markers of cognitive and memory impairment as well as Parkinson’s disease or multiple system atrophy (Bucerius et al., 2012). [^11^C]DASB, [^11^C]McN 5652 and [^18^F]FMe-McN5652 have been shown to bind serotonin (5-hydroxytryptamine, 5-HT) receptors and the 5-HT transporter. These transporters are dysregulated in affective disorders and shall be valuable structures for the assessment of activity of antidepressants. In the midbrain and amygdala of patients with major depressive disorder lower 5-HT transporter binding can be assessed with tracers such as [^11^C]McN5652 (Parsey et al., 2006). Furthermore, this group of tracers is capable to determine abnormalities in both serotonin transporter and dopamine transporter binding as a result of the abnormalities in the neurotransmitter systems of the brains of autistic individuals (Hesse et al., 2012). Due to the salient role of opioid systems in neuropathic pain (Maarrawi et al., 2013), PET imaging of opioid receptors facilitates the understanding of the molecular and cellular mechanisms of pain generation and assists the use and advancement of pain medication, spinal cord stimulation and spinal infusion pumps. The opioid receptor binding tracer [^11^C]diprenorphine reveals reduced opioid receptor densities in neuropathic pain patients. Another tracer that binds to the GABA-receptors [^11^C]flumazenil has been used to study increased cortical excitability in focal epilepsy (Szelies et al., 2002).

The pervasive search for drug treatments targeting Alzheimer’s disease has stimulated tracers to provide an intimate link to the diagnostics of this potential blockbuster market. Radiotracers binding the plaque forming proteins beta-amyloid- and tau-protein represent a straightforward approach for this disease. Subsequently, a series of different tracers ofthe Pittsburgh compound, e.g., florbetaben and flutemetamol have recently been approved for the detection of Alzheimer’s disease related protein plaques and the accompanying potential of staging this disease (Nordberg et al., 2010). Interestingly, a radiolabeled derivative of the dye thioflavin T was shown to bind the amyloid protein deposits in the brains of patients suffering from Alzheimer’s disease (Kreisl et al., 2013). Other examples for the various PET tracers for neuroimaging are 6-[^18^F]fluoro-L-DOPA that allows the imaging of presynaptic dopamine and can be used to determine dopamine turnover in Parkinson patients (Sossi et al., 2002).

The study of tracers that map the regional differences of blood flow represents a different approach to visualize pathological changes and to gain function information of the brain. ^15^O-H_2_O is the gold standard for this purpose (Grüner et al., 2012). This is complemented by 2-[^18^F]fluoro-2-deoxy-D-glucose (FDG), the working horse of modern molecular imaging (de Leon et al., 2001). In addition, to provide perfusion data, FDG allows conclusions about neuronal activity based on the energy metabolism. FDG is taken up by glucose transporters and trapped inside the cell as its 6-phosphate as a result of phosphorylation by the enzyme hexokinase. As the progression of Alzheimer’s disease is accompanied by decreased brain metabolism, FDG-PET can be used to diagnose its early stages and for the differentiation from other processes of dementia (Mosconi et al., 2008). Due to the short half-lives of ^11^C (20 min) and ^18^F (108 min) very small amounts of the tracer are required to obtain the appropriate signal intensity and the total radiation exposure is comparable to the one obtained by whole body CT scans. The short half lives in turn entail the on demand synthesis of the radiotracers ideally with a cyclotron in close proximity to the PET imaging facility. The effective doses are continuously reduced by using tracers with short physical and biological half-lives, by minimizing the activity injected and by the ongoing enhancement of the sensitivity of PET scanners.

## Prinicples of Magnetic Resonance Imaging

While PET imaging allows the assessment of neurotransmitter concentrations in the brain, it is not suitable to reveal micro- and macroscopic structural aberrations in the white and gray matter. In addition, PET cannot be used to detect rapid changes in brain activation. Moreover, despite the enormous achievement of PET imaging there is always a concern about the radiation risks involved in this modality. Magnetic resonance spectroscopy (MRS) represents an alternative as endogenous compounds involved in the biochemistry of the brain are detected. The resonance frequency of protons depends on their chemical structure and MRS measures the concentration of marker molecules such as methionine or lipids (van der Graaf, 2010). Again, the performance of the imaging modality is limited by the contrast enhancing molecules: the concentrations of significant compounds such as neurotransmitters in the brain are far below the detection limit. In contrast, the bulk of the signal in magnetic resonance imaging comes from free water and this is present in abundance. The sizeable differences varying from 99 percent in the CSF over about 80 percent in gray matter to 70 percent in white matter are the basis for the excellent soft tissue contrast observed in MRI images of the brain (Neeb et al., 2006). Due to its high spatial resolution and the possibility to detect chances in brain morphology without the application of tracers, MRI has become the clinically preferred method of imaging soft tissues, such as the brain.

While in principle, MRI can be conducted without the application of contrast agents to the patient, for diagnostic purposes, e.g., early detection of strokes, contrast agents have to be applied. At first glance the sensitivity of MRI is similar to that of CT and high concentrations of the contrast agent should be required. But there is a fundamental difference between the contrast agents used in CT and MRI. The currently used MRI contrast agents (mostly gadolinium complexes that cause T1-shortening) are working indirectly by affecting the relaxation of water molecules in their coordination sphere. Due to the high exchange rate of water in the sphere of gadolinium complexes a large number of water molecules are relaxed within one scan (typically 2–3 s). As a result, an apparent several thousand fold amplification of the effect is observed. Consequently, a huge variety of MRI contrast agents have been developed (van der Graaf, 2010).

After evidence for a link between nephrogenic systemic fibrosis (NFS) and gadolinium contrast agents had been described (Cowper et al., 2000), the risk of NSF has become an important issue in radiology. There is a broad consensus that the residual risk caused by gadolinium contrast agents only exists for patients with impaired kidney function. Even though NFS was associated with only an infinitesimal insignificant number of the patients diagnosed with gadolinium contrast agents, their link to the risk of NSF has greatly affected the development of MRI contrast agents and the approval of novel MRI contrast agents by the FDA has collapsed since. This is an unbearable situation: due to their elemental role in patient care, MRI contrast agents hold invaluable benefit for today’s health care. A discussion of the obligation to strike between the benefits of novel contrast agents and the risk of their widespread application (please consider that out of necessity the formerly approved contrast agents are still used!) is beyond the scope of this article.

## Functional Magnetic Resonance Imaging

Given the difficulties described for the development of specific MRI contrast agents, fMRI was developed in due course. As discussed above, the success of MRI is in great part due to the fact that an endogenous contrast agent is detected at high sensitivity. fMRI provides further characteristics that contribute to this exceptional performance. In the preferred application of the fMRI technique the iron in deoxyhemoglobin acts as a paramagnetic contrast agent similarly as gadolinium in the common MRI contrast agents. The blood of adults contains about 750 g of hemoglobin, an amount hardly to be attained with endogenous contrast agents. In deoxygenated hemoglobin the iron is paramagnetic as it has four unpaired electrons. Upon oxidation, hemoglobin loses its contrast enhancing properties. Consequently, the deoxyhemoglobin/oxyhemoglobin ratio defines the signal intensity. The so called blood oxygen level dependent (BOLD)-effect can be traced back to neural activity (rather than spiking of neurons), probably mainly due to increased uptake of glutamate in astrocytes (Logothetis et al., 2001; Raichle, 2001).

Several elementary features contribute to the unmatched performance of fMRI for neurological applications: (a) its clinical convenience—fMRI is performed with the standard MRI scanners and does not require the injection of contrast agents, but is achieved due to the measurement of an endogenous contrast agent that is present at high concentration in the brain; (b) the fact that the contrast agent is functional and responsible to stimuli; (c) the fact that fMRI it is not associated with the use of radioactive probes provides a high ease of repeatability and therefore makes longitudinal studies of subjects possible; and (d) the possibility to study not only alterations in brain morphology that affect activation of a certain region, but also its connectivity to other brain regions.

## Applications for (f)MRI

Nowadays, MRI scans of the brain are standard in clinical care in neurologic and psychiatric hospitals. Neurological, as well as vascular diseases of the brain can be easily detected and help guiding diagnostics and appropriate therapies. In addition, fMRIs can be acquired prior to brain surgeries to allow the surgeon an individualized anatomical mapping of visual and motoric functions of the patient.

**Figure 1:**
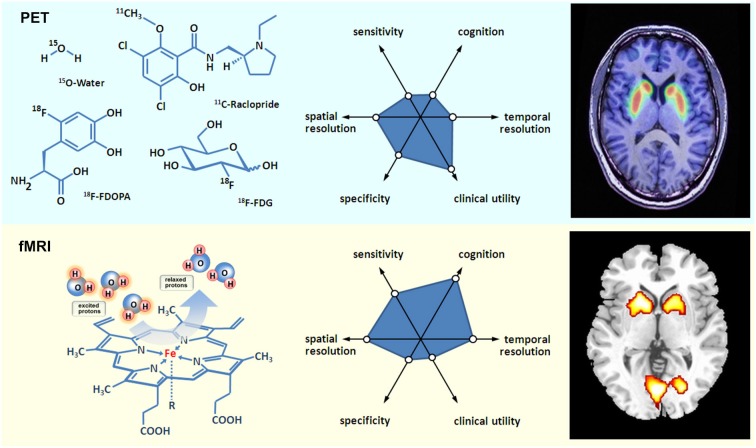
**Basic aspects of PET and fMRI.** Left: chemical structures of common PET tracers used in neurological imaging and illustration of the induction of relaxation by hemoglobin, Middle: General characteristics of PET versus fMRI, Right: Typical PET and fMRI images (both single subjects).

Moreover, fMRI has a prominent role in neurologic and psychiatric brain imaging research. For the sake of brevity, the following illustration of MRI and fMRI for psychiatric research will be exemplarily illustrated for schizophrenia. MRI studies on schizophrenia show a volume loss of brain tissue, specially an enlargement of the ventricles, replicating earlier CT studies, making it one of the most robust findings in MRI research (Shenton et al., 2001). The first fMRI studies on schizophrenia revealed dorsolateral prefrontal cortex and temporal cortex as neural substrate of deficient cognition (Kindermann et al., 1997). More recently, the focus has shifted to (a) the investigation of socio-emotional processing in schizophrenia, as well as (b) to the investigation of resting state to detect task independent alterations in the connectivity of brain networks (Zhou et al., 2007) that both give according evidence for prefrontal and temporal alterations. Consequently, an additional achievement of fMRI becomes obvious: fMRI does not only allow the detection of altered brain activation, but also of altered brain connectivity, indicating disturbed communication of brain areas. And indeed schizophrenia that has long been described as a disorder of disconnectivity, could be empirically linked to altered functional (Meyer-Lindenberg et al., 2001), as well as structural (Burns et al., 2003) prefrontal-temporal communication by means of fMRI, and diffusion tensor imaging (DTI) that gives evidence for myelin loss of white matter tracks. Another modality of high interest in MRI scanners is MRS. By allowing the investigation of glutamate in the prefrontal cortex of patients with schizophrenia, MRS helps to solve another puzzle of the cognitive deficits in schizophrenia, (Poels et al., 2014). Moreover, attempts to investigate molecular processes in the brain by means of fMRI are the imaging genetics studies that combine genetic information for e.g., a certain SNiP that is known to influence the availability of dopamine in the frontal cortex with brain activation (Mier et al., 2010). However, using MRS the concentration of only few neurotransmitters can be estimated, and imaging genetics studies might neglect epigenetic effects, while PET has a wide range of possible molecular targets in the brain that can be directly assessed. Notwithstanding the additional advantages and necessities of MRS and imaging genetics studies, a combination of (f)MRI and PET would allow a direct comprehensive assessment of the functioning of individual brain regions, as well as the interplay on the level of the whole brain (see Figure 1 for a schematic comparison of PET and (f)MRI).

## Hybrid PET/fMRI

Generally, in PET the advances are driven by the development of selective radioligands (i.e., tracers targeting Alzheimer’s disease) and in fMRI the development of the instrumentation (i.e., the implementation of high-field MRI into clinical applications), as well as the analysis methods (i.e., advances methods for connectivity analyses, such as dynamic causal modelling).

However, improvements of the specificity of molecular imaging probes invariably go along with the loss of effectual resolution as the orientation guide—the accumulation in the non-target tissue is decreased. Hence, bimodal imaging techniques that combine the outstanding molecular imaging probabilities of PET with imaging techniques with a better spatial resolution were developed. PET/CT is the prime example for such a bimodal imaging technique as the anatomical information gained at high resolution by CT synergistically matches the functional information of PET imaging. Due to the lack of tissue that provides significant attenuation of X-rays in the brain, PET/CT images of the brain do not significantly benefit from the CT-portion of the fused images. Since MRI has excellent soft tissue contrast, MRI should be perfect to navigate the PET information in the brain and high hopes are placed into the clinical outcome of the PET/MRI scanners that currently emerge. Hence, despite the exceptional technical challenges that had to be mastered (Pichler et al., 2008), PET/MRI hybrid systems have been developed. These PET/MRIs do not only allow to acquire PET and MRI with the same machine, but to acquire them simultaneously, because there is more to this combination than the convergence of anatomical and molecular resolution. While the determination of increases in neural activity and metabolism by the BOLD effect at first glance resembles static tracers that track the hemodynamics (i.e., CT and MRI blood pool contrast agents), the BOLD effect gives insight into the site of oxygen consumption and thus metabolic information. Hemoglobin is not able to penetrate the blood brain barrier but as it is triggered by the readily diffusible molecule oxygen, it virtually reports on metabolism beyond the blood brain barrier. Hence, this is the first time that it is possible to directly and simultaneously investigate the activity of neurotransmitters along with the activity and connectivity of a brain region. This combined assessment will give rise to a next generation of understanding brain function in healthy persons and patients with mental illnesses, and thus gives rise to high promise on the way to personalized therapies—the great white hope in medicine.

## Conclusions

Despite the complexity of the brain function, advances in brain imaging techniques, in particular PET and MRI have boosted the research in the structure and function of the human brain. MRI has become the main modality for clinical neuroimaging. While economical restrictions and unbalanced safety concerns halt the development of novel tracers, fMRI hemodynamic based strategies have strongly advanced the progress of neuroimaging. The molecular imaging tracers (predominantly radiotracers) for PET provide the basis not only for a better understanding of molecular processes in the brain, but also for the stratification procedures essential for the safe development of individualized therapies. The directly combined application of BOLD fMRI measuring brain function with PET radiolabeled reporters that map the distribution and function of receptors synergistic will form the basis of a new area in today’s rapidly increasing insight into brain function.

## Conflict of Interest Statement

The authors declare that the research was conducted in the absence of any commercial or financial relationships that could be construed as a potential conflict of interest.

## References

[B1] Bucerius J. Manka C. Schmaljohann J. Mani V. Gündisch D. Rudd J. H. F.. (2012). Feasibility of [18F]-2-Fluoro-A85380-PET imaging of human vascular nicotinic acetylcholine receptors *in vivo*. JACC Cardiovasc. Imaging 5, 528–536. 10.1016/j.jcmg.2011.11.024 22595161 PMC3623271

[B2] Burns J. Job D. Bastin M. E. Whalley H. Macgillivray T. Johnstone E. C.. (2003). Structural disconnectivity in schizophrenia: a diffusion tensor magnetic resonance imaging study. Br. J. Psychiatry 182, 439–443. 10.1192/bjp.02.396 12724248

[B3] Catana C. Drzezga A. Heiss W. D. Rosen B. R. (2012). PET/MRI for neurologic applications. J. Nucl. Med. 53, 1916–1925. 10.2967/jnumed.112.105346 23143086 PMC3806202

[B4] Cowper S. E. Robin H. S. Steinberg S. M. Su L. D. Gupta S. LeBoit P. E. (2000). Scleromyxoedema-like cutaneous diseases in renal-dialysis patients. Lancet 356, 1000–1001. 10.1016/s0140-6736(00)02694-5 11041404

[B5] de Leon M. J. Convit A. Wolf O. T. Tarshish C. Y. DeSanti S. Rusinek H.. (2001). Prediction of cognitive decline in normal elderly subjects with 2-[(18)F]fluoro-2-deoxy-D-glucose/poitron-emission tomography (FDG/PET). Proc. Natl. Acad. Sci. U S A 98, 10966–10971. 10.1073/pnas.191044198 11526211 PMC58582

[B6] Grüner J. M. Paamand R. Kosteljanetz M. Broholm H. Højgaard L. Law I. (2012). Brain perfusion CT compared with (1)(5)O-H(2)O PET in patients with primary brain tumours. Eur. J. Nucl. Med. Mol. Imaging 39, 1691–1701. 10.1007/s00259-012-2173-1 22736199 PMC3464373

[B7] Hesse S. Brust P. Mäding P. Becker G. A. Patt M. Seese A.. (2012). Imaging of the brain serotonin transporters (SERT) with 18F-labelled fluoromethyl-McN5652 and PET in humans. Eur. J. Nucl. Med. Mol. Imaging 39, 1001–1011. 10.1007/s00259-012-2078-z 22349718

[B8] Kindermann S. S. Karimi A. Symonds L. Brown G. G. Jeste D. V. (1997). Review of functional magnetic resonance imaging in schizophrenia. Schizophr. Res. 27, 143–156. 10.1016/s0920-9964(97)00063-7 9416644

[B9] Kreisl W. C. Lyoo C. H. McGwier M. Snow J. Jenko K. J. Kimura N.. (2013). *In vivo* radioligand binding to translocator protein correlates with severity of Alzheimer’s disease. Brain 136, 2228–2238. 10.1093/brain/awt145 23775979 PMC3692038

[B10] Logothetis N. K. Pauls J. Augath M. Trinath T. Oeltermann A. (2001). Neurophysiological investigation of the basis of the fMRI signal. Nature 412, 150–157. 10.1038/35084005 11449264

[B11] Maarrawi J. Peyron R. Mertens P. Costes N. Magnin M. Sindou M.. (2013). Brain opioid receptor density predicts motor cortex stimulation efficacy for chronic pain. Pain 154, 2563–2568. 10.1016/j.pain.2013.07.042 23900133

[B12] Meyer-Lindenberg A. Poline J. B. Kohn P. D. Holt J. L. Egan M. F. Weinberger D. R.. (2001). Evidence for abnormal cortical functional connectivity during working memory in schizophrenia. Am. J. Psychiatry 158, 1809–1817. 10.1176/appi.ajp.158.11.1809 11691686

[B13] Mier D. Kirsch P. Meyer-Lindenberg A. (2010). Neural substrates of pleiotropic action of genetic variation in COMT: a meta-analysis. Mol. Psychiatry 15, 918–927. 10.1038/mp.2009.36 19417742

[B14] Mortelé K. J. Oliva M. R. Ondategui S. Ros P. R. Silverman S. G. (2005). Universal use of nonionic iodinated contrast medium for CT: evaluation of safety in a large urban teaching hospital. AJR Am. J. Roentgenol. 184, 31–34. 10.2214/ajr.184.1.01840031 15615946

[B15] Mosconi L. Tsui W. H. Herholz K. Pupi A. Drzezga A. Lucignani G.. (2008). Multicenter standardized 18F-FDG PET diagnosis of mild cognitive impairment, Alzheimer’s disease and other dementias. J. Nucl. Med. 49, 390–398. 10.2967/jnumed.107.045385 18287270 PMC3703818

[B16] Neeb H. Zilles K. Shah N. J. (2006). Fully-automated detection of cerebral water content changes: study of age- and gender-related H2O patterns with quantitative MRI. Neuroimage 29, 910–922. 10.1016/j.neuroimage.2005.08.062 16303316

[B17] Nordberg A. Rinne J. O. Kadir A. Långström B. (2010). The use of PET in Alzheimer disease. Nat. Rev. Neurol. 6, 78–87. 10.1038/nrneurol.2009.217 20139997

[B18] Parsey R. V. Oquendo M. A. Ogden R. T. Olvet D. M. Simpson N. Huang Y. Y.. (2006). Altered serotonin 1A binding in major depression: a [carbonyl-C-11]WAY100635 positron emission tomography study. Biol. Psychiatry 59, 106–113. 10.1016/j.biopsych.2005.06.016 16154547

[B19] Pichler B. J. Judenhofer M. S. Wehrl H. F. (2008). PET/MRI hybrid imaging: devices and initial results. Eur. Radiol. 18, 1077–1086. 10.1007/s00330-008-0857-5 18357456

[B20] Poels E. M. Kegeles L. S. Kantrowitz J. T. Javitt D. C. Lieberman J. A. Abi-Dargham A.. (2014). Glutamatergic abnormalities in schizophrenia: a review of proton MRS findings. Schizophr. Res. 152, 325–332. 10.1016/j.schres.2013.12.013 24418122 PMC3951718

[B21] Raichle M. E. (2001). Cognitive neuroscience. Bold insights. Nature 412, 128–130. 10.1038/35084300 11449247

[B22] Shenton M. E. Dickey C. C. Frumin M. McCarley R. W. (2001). A review of MRI findings in schizophrenia. Schizophr. Res. 49, 1–52. 10.1016/s0920-9964(01)00163-3 11343862 PMC2812015

[B23] Siessmeier T. Zhou Y. Buchholz H. G. Landvogt C. Vernaleken I. Piel M.. (2005). Parametric mapping of binding in human brain of D2 receptor ligands of different affinities. J. Nucl. Med. 46, 964–972. 15937307

[B24] Sossi V. de La Fuente-Fernández R. Holden J. E. Doudet D. J. McKenzie J. Stoessl A. J.. (2002). Increase in dopamine turnover occurs early in Parkinson’s disease: evidence from a new modeling approach to PET 18 F-fluorodopa data. J. Cereb. Blood Flow Metab. 22, 232–239. 10.1097/00004647-200202000-00011 11823721

[B25] Szelies B. Sobesky J. Pawlik G. Mielke R. Bauer B. Herholz K.. (2002). Impaired benzodiazepine receptor binding in peri-lesional cortex of patients with symptomatic epilepsies studied by [(11)C]-flumazenil PET. Eur. J. Neurol. 9, 137–142. 10.1046/j.1468-1331.2002.00338.x 11882054

[B26] van der Graaf M. (2010). *In vivo* magnetic resonance spectroscopy: basic methodology and clinical applications. Eur. Biophys. J. 39, 527–540. 10.1007/s00249-009-0517-y 19680645 PMC2841275

[B27] Zhou Y. Liang M. Jiang T. Tian L. Liu Y. Liu Z.. (2007). Functional dysconnectivity of the dorsolateral prefrontal cortex in first-episode schizophrenia using resting-state fMRI. Neurosci. Lett. 417, 297–302. 10.1016/j.neulet.2007.02.081 17399900

